# Draft Genome Sequence of *Klebsiella pneumoniae* Strain KP-1

**DOI:** 10.1128/genomeA.01082-13

**Published:** 2013-12-19

**Authors:** Kai Wei Kelvin Lee, Krithika Arumugam, Rikky Wenang Purbojati, Qi Xiang Martin Tay, Rohan Benjamin Hugh Williams, Staffan Kjelleberg, Scott A. Rice

**Affiliations:** Singapore Centre on Environmental Life Sciences Engineering, Nanyang Technological University, Singaporea; School of Biological Sciences, Nanyang Technological University, Singaporeb; Centre for Marine Bio-Innovation and School of Biotechnology and Biomolecular Sciences, University of New South Wales, Sydney, Australiac

## Abstract

*Klebsiella pneumoniae* is ubiquitous in the environment and is a member of a three-species biofilm model. We compared the genome sequence of an environmental isolate, *K. pneumoniae* strain KP-1, to those of two clinical strains (NTUH-K2044 and MGH 78578). KP-1 possesses strain-specific prophage sequences that distinguish it from the clinical strains.

## GENOME ANNOUNCEMENT

*Klebsiella pneumoniae* is ubiquitous in the environment, where it is involved in nitrogen fixation (1). It also causes bovine mastitis (2) and nosocomial infections in humans (3). It coexists with *Pseudomonas aeruginosa* and *Pseudomonas protegens* in metalworking fluids and the gut of the silk moth *Bombyx mori* (4, 5). A reproducible, mixed-species biofilm model comprising *K. pneumoniae* KP-1, *P. aeruginosa* PAO1, and *P. protegens* Pf-5 was thus developed to study bacterial interspecies interactions and their effects on biofilm development and fitness (6). While the complete genome sequences of PAO1 and Pf-5 were available to facilitate “omics” studies such as transcriptomics, KP-1 is a newly isolated environmental isolate, and its genome has not been sequenced previously.

The strain was shotgun sequenced on a 454 GS-FLX sequencing platform (Roche, Basel, Switzerland) and an Illumina MiSeq benchtop sequencer (Illumina, CA, USA). The reads from both platforms were trimmed, and *de novo* assembly was performed using Newbler v2.6 (Roche). With 1,673,246 and 1,840,620 reads from the 454 GS-FLX and MiSeq sequencing platforms, respectively, 24 contigs with a total length of 5,131,085 bp and an average GC content of 57.6% were assembled. The open reading frames (ORFs) were predicted using Glimmer v3.02 (7) and annotated by performing BLASTX analysis (*E* value <10^-3^, >80% identity) against the nonredundant protein sequence database of the National Center for Biotechnology Information. The tRNAs were predicted using tRNAscan-SE v1.3.1 (8), while rRNA sequences were identified by RNAmmer v1.2 (9). A total of 4,587 ORFs were annotated in addition to 73 tRNAs, five 5S rRNAs, and one 23S rRNA. Genomic comparisons to two clinical isolates, *K. pneumoniae* strain NTUH-K2044 (GenBank accession number AP006725) and strain MGH 78578 (GenBank accession number CP000647), by mGenomeSubtractor v1.3 (*H* value <0.42) (10) showed that KP-1 possesses several strain-specific genes that encode phage-related proteins such as tail proteins, capsid proteins, terminases, and integrases. Differences between the prophage sequences of strain NTUH-K2044 and strain MGH 78578 were previously reported and suggested to be markers that could be used to distinguish the various lineages of *K. pneumoniae* (11). The present study further suggests that prophage sequences could be the ideal evolutionary markers for identifying the different lineages of *K. pneumoniae*.

### Nucleotide sequence accession numbers.

This whole-genome shotgun project has been deposited in DDBJ/EMBL/GenBank under the accession number AVNZ00000000. The version described in this paper is version AVNZ01000000.
